# The role of beta-blocker drugs in critically ill patients: a SIAARTI expert consensus statement

**DOI:** 10.1186/s44158-023-00126-2

**Published:** 2023-10-23

**Authors:** Fabio Guarracino, Andrea Cortegiani, Massimo Antonelli, Astrid Behr, Giandomenico Biancofiore, Alfredo Del Gaudio, Francesco Forfori, Nicola Galdieri, Giacomo Grasselli, Gianluca Paternoster, Monica Rocco, Stefano Romagnoli, Salvatore Sardo, Sascha Treskatsch, Vincenzo Francesco Tripodi, Luigi Tritapepe

**Affiliations:** 1 Cardiothoracic and Vascular Anesthesia and Intensive Care, Anesthesia and Resuscitation Department, Azienda Ospedaliero Universitaria Pisana, Pisa, Italy; 2 Department of Surgical, Oncological and Oral Science (Di.Chir.On.S.), University of Palermo, Palermo, Italy; 3 Department of Anesthesia, Intensive Care and Emergency, Policlinico Paolo Giaccone, University of Palermo, 90127 Palermo, Italy; 4 Department of Emergency, Anesthesiological and Resuscitation Sciences, Policlinico Universitario A. Gemelli IRCCS, Rome, Italy; 5 Operative Unit of Anesthesia and Resuscitation, Hospital of Camposampiero, Padua, Italy; 6 Anesthesia and Resuscitation Transplants, Department of Medical Pathology Surgical, Molecular and Critical Area, University of Pisa, Pisa, Italy; 7 Emergency Department, Casa Sollievo Della Sofferenza, San Giovanni Rotondo, Italy; 8 Anesthesia and Intensive Care, Anesthesia and Resuscitation Department, Azienda Ospedaliero Universitaria Pisana, Pisa, Italy; 9 General Cardiac Surgery Unit, Critical Area Department, Ospedale Dei Colli, Naples, Italy; 10 Department of Anesthesia, Resuscitation and Emergency, IRCCS Ca’ Granda Foundation, Ospedale Maggiore Policlinico, Milan, Italy; 11 Department of Medical-Surgical and Transplant Pathophysiology, University of Milan, Milan, Italy; 12 Cardiovascular Anesthesia and ICU, San Carlo Hospital, Potenza, Italy; 13 Department of Surgical and Medical Science and Translational Medicine, Sapienza University of Rome, Rome, Italy; 14 Anesthesia and Intensive Care Section, Department of Health Sciences, University of Florence, Florence, Italy; 15 Department of Anesthesia and Intensive Care, Careggi University Hospital, Florence, Italy; 16 Department of Medical Sciences and Public Health, University of Cagliari, Cagliari, Italy; 17 Department of Anesthesiology and Intensive Care Medicine, Charité Universitätsmedizin Berlin, Freie Universität and Humboldt-Universität Zu Berlin, Hindenburgdamm 30, 12203 Berlin, Germany; 18 Anesthesia and Intensive Care Unit, Department of Surgery, University Hospital “Gaetano Martino”, Messina, Italy; 19 Anesthesia and Resuscitation Unit, San Camillo-Forlanini Hospital, Sapienza University, Rome, Italy

**Keywords:** β-blockers, Critically ill patient, ICU, Tachyarrhythmia, Sepsis, Adults

## Abstract

**Background:**

The role of β-blockers in the critically ill has been studied, and data on the protective effects of these drugs on critically ill patients have been repeatedly reported in the literature over the last two decades. However, consensus and guidelines by scientific societies on the use of β-blockers in critically ill patients are still lacking.

The purpose of this document is to support the clinical decision-making process regarding the use of β-blockers in critically ill patients. The recipients of this document are physicians, nurses, healthcare personnel, and other professionals involved in the patient’s care process.

**Methods:**

The Italian Society of Anesthesia, Analgesia, Resuscitation and Intensive Care (SIAARTI) selected a panel of experts and asked them to define key aspects underlying the use of β-blockers in critically ill adult patients. The methodology followed by the experts during this process was in line with principles of modified Delphi and RAND-UCLA methods. The experts developed statements and supportive rationales in the form of informative text. The overall list of statements was subjected to blind votes for consensus.

**Results:**

The literature search suggests that adrenergic stress and increased heart rate in critically ill patients are associated with organ dysfunction and increased mortality. Heart rate control thus seems to be critical in the management of the critically ill patient, requiring careful clinical evaluation aimed at both the differential diagnosis to treat secondary tachycardia and the treatment of rhythm disturbance. In addition, the use of β-blockers for the treatment of persistent tachycardia may be considered in patients with septic shock once hypovolemia has been ruled out. Intravenous application should be the preferred route of administration.

**Conclusion:**

β-blockers protective effects in critically ill patients have been repeatedly reported in the literature. Their use in the acute treatment of increased heart rate requires understanding of the pathophysiology and careful differential diagnosis, as all causes of tachycardia should be ruled out and addressed first.

**Supplementary Information:**

The online version contains supplementary material available at 10.1186/s44158-023-00126-2.

## Background

Critically ill patients admitted to an intensive care unit (ICU) may be affected by different degrees of sympathetic overflow [1–4] secondary to the primary acute disease (i.e., shock, trauma, infection) and often present with preexisting cardiovascular comorbidities. The role of β-blockers in critically ill patients has been studied, and data on the protective effects of these drugs on the critically ill have been repeatedly reported in the literature over the last two decades.

β-blockers exert their effects through several subtypes of G-protein-coupled β-adrenergic receptors [5] expressed on the surface of cell membranes almost ubiquitously in the human body.

Generally, β-blockers interfere with catecholamines and sympathomimetics by preventing and/or modulating the β-adrenergic responses. The clinical effects depend on the subtypes of receptors a β-blocker binds to and their locations (Table 1).

**Table 1:** Pharmacokinetics and pharmacodynamic properties of intravenous β-blockers

Drug	Onset (min)	Max. elimination half-life (min)	Receptors	Cardio-selectivity (β1–β2)	Metabolization	HR	MAP
**Metoprolol**	**1–3**	**420**	**β1**	**3**	**Cytochrome P2D6 (Leber)**	**↓**	**↓**
**Labetalol**	**2–5**	**120–480**	**β1–β2–α1**	**No**	**By the liver resulting in an inactive glucuronide conjugate**	**↓**	**↓↓**
**Propanolol**	** < 5**	**360–600**	**1–β2**	**No**	**Cytochrome P2D6**	**↓**	**↓**
**Esmolol**	**2**	**9**	**β1**	**30**	**ERY esterases**	**↓↓**	**↓**
**Landiolol**	**1**	**4**	**β1**	**250**	**Pseudocholinesterases**	**↓↓**	** ↔ /↓**

Legend: *Max* Maximum, *min* Minutes, *HR* Heart rate, *MAP* Mean arterial pressure

Based on their pharmacodynamics, β-blockers are currently indicated for treating systemic arterial hypertension, tachyarrhythmias, and heart failure [6]. In addition, β-blockers decrease blood pressure by reducing afterload. This in turn together decreases myocardial oxygen consumption and improves myocardial perfusion as well as stroke volume (SV). They also show effective control of sympathetic activation, and their proven efficacy for treating rhythm alterations [7] represents a strong pathophysiological rationale to consider such treatment in the management of critically ill patients suffering from acute tachyarrhythmias.

However, consensus and guidelines by scientific societies on the use of β-blockers in critical illness are still lacking, and guidelines on management of septic patients have never recommended their use in this setting. To fill this gap, a group of expert intensivists selected by the *Italian Society of Anesthesia, Analgesia, and Intensive Care (SIAARTI)* wrote this good clinical practice document on the use of beta-blockers in critically ill patients.

## Methodology

The expert panel members were selected by the two project coordinators (F. G., L. T.) based on evidence, clinical, and scientific experience on the subject on behalf of SIAARTI. After an initial meeting to define the methodology, the different topics were assigned to one or more panel members, based on their respective skills, as follows:

- Evaluate the available evidence.
- Produce statements and supporting rationales in the form of an explanatory text.

The overall list of statements was submitted to a vote, according to the method, to express the degree of consensus.

The methodological path of the document was outlined by a methodologist (A. C.) and was based on the auditing principles of scientific literature and the modified Delphi method [8]. More in detail, the literature review was conducted by two subject matter experts with no defined time limit, on PubMed, using MeSH words [1] (E-Table 1 — Additional File 1).

The panel of experts defined four clinical questions (CQ) that were presented and voted during an online scoping workshop. Following up the definition of the questions, the two subject matter experts defined a dedicated literature search strategy. The two experts then selected the relevant literature from the list generated by the search and correlated each chosen paper to one or more of the four clinical questions. The list of chosen papers was then submitted to the panel for review (Additional File 2).

The types of papers included in the search were as follows: randomized controlled trials, systematic reviews, meta-analysis, guidelines, non-randomized controlled trials, narrative reviews, position papers, and experimental studies. Papers with a language different from English were excluded, as were conference proceedings, case reports, and case series. The search was conducted, and the final reports were generated following the principles of PRISMA 2020 [9] (Fig. 1 in the Additional File 1). The panelists, using the report from the subject matter experts and their competencies, drafted a list of statements and rationales that they then put for vote on a secret ballot. The entire panel (with the exclusion of the search specialist and methodologist) took part in the blind vote. The methodology dictated a maximum of two possible rounds of voting online.

The opinion was expressed using an ordinal Likert scale, according to the RAND-UCLA method (minimum score, 1 = completely disagree; maximum score, 9 = completely agree). This scale was divided into 3 sections: 1–3 implied refusal/disagreement (“inappropriate”), 4–6 implied (“uncertainty”), and 7–9 implied agreement/support (“appropriateness”) [10].

A consensus was reached when as follows:

1. At least 75% of the respondents (excluding the methodologist and the search specialist) assigned a score between 1–3, 4–6, or 7–9, which meant refusal, uncertainty, and agreement of the statement, respectively.
2. The median score was within the same range. The type of consensus was determined by the positioning of the median. It was not necessary to run the second Delphi round, as all statements reached consensus at the first round. The results of the votes were reported in a tabulated form. The full version of the Italian document issued by the Italian Society of Anesthesia, Analgesia, Resuscitation, and Intensive Care (SIAARTI) was published in April 2023 and is freely available on the society’s website in Italian (https://www.siaarti.it/news/1527682).

### Question I.  What is the rationale for correcting tachycardia in the critically ill patients?

In critically ill patients, the acute state of illness often increases myocardial and tissue oxygen consumption (VO_2_) in general and causes profound adrenergic activation. Adrenergic hypertonicity is one of the protagonists of the complex neuroendocrine response that the patient exhibits when oxygen delivery (DO_2_) and his VO_2_ lose the optimal ratio [11, 12]*.* The continuation of this rapid compensation mechanism, even if the causes that may have triggered it are treated (e.g., anemia, hypovolemia, pain, and hyperthermia), can favor hypoperfusion secondary to vasoconstriction and an inevitable increase in myocardial VO_2_ linked to tachycardia or heart failure [3, 4].

This scenario is commonly accompanied by an increase in heart rate, which can take the form of sinus tachycardia or lead to tachyarrhythmias possibly associated with hemodynamic instability (hypotension and/or hypoperfusion).

This excessive adrenergic stress and increased heart rate have been associated with organ dysfunction and increased mortality [13–15]*.*

In this pathophysiological scenario, in light of literature data, heart rate control assumes an important role in the management of the critically ill patient, requiring careful clinical evaluation aimed at both the differential diagnosis of secondary tachycardia and the treatment of the rhythm alteration.

Physiologically, the main compensation mechanisms involve the release of endogenous catecholamines with sympathetic hyperstimulation. The use of exogenous catecholamines may also be necessary for the treatment of hemodynamic instability. However, in both cases, excessive adrenergic stimulation is related to organ damage with worsening of the outcome and increased mortality.

The diagnostic framework of tachycardia requires the analysis of the electrocardiographic tracing and the appropriate integration of this within an echocardiographic examination [16]. It is defined as a heart rate (HR) greater than 100 bpm [12, 17].

It can originate as follows:

- At sinus node and is defined as sinus tachycardia;
- At the ventricular level and is defined as ventricular tachycardia, usually of short duration and accompanied by important hemodynamic alterations;
- From supraventricular origin and is generally represented by an altered electrical activity in the atrial chambers and is due either to the re-entry mechanism or to an increase in automaticity

In the latter case, it can manifest itself as follows:

- Atrial flutter with regular sawtooth waves at a rate of 180–250/min;
- Tachyarrhythmia absoluta with atrial fibrillation waves replacing P waves followed by abnormal QRS complexes;
- Ventricular tachycardia with irregular RR intervals and QRS complexes with abnormal duration and morphology and not associated with a previous P wave.

Echocardiography performed in order to diagnose the origin of tachycardia and to guide the appropriateness of beta-blockers administration should focus on both systolic and diastolic function, as well as on volume status. The calculation of the ventriculo-arterial coupling, a more complex evaluation, can be added to this echocardiographic evaluation to understand the efficiency of the cardiovascular system [18] and to reassess the patient’s physiology after treatment.

### Question II. What is the rationale for using a β-blocker to correct tachycardia in critical patients?

#### In the critically ill patient, tachycardia control should be achieved with the use of β-blocking drugs

A recent meta-analysis of 11 studies, including 2103 critically ill patients, showed a significant reduction in mortality (risk ratio 0.65, 95% *CI* 0.53–0.79; *P* < 0.0001) in patients treated with β-blockers compared to controls [12]. However, it is important to note that this systematic review and meta-analysis included a diverse set of studies, with the majority of them focusing on patients with myocardial ischemia or those undergoing cardiac surgery. As a result, it becomes challenging to make definitive conclusions regarding the observed differences in mortality.

Another recent retrospective study of 204,981 patients undergoing major abdominal surgery, conducted with propensity score analysis, found no differences in the incidence of postoperative stroke among patients receiving β-blocker in chronic therapy (odds ratio, 0.86; 95% *CI*, 0.65 to 1.15; *P* = 0.901) and patients in whom the drug was started within 60 days before surgery (odds ratio, 0.90; 95% *CI*, 0.31 to 2.04; *P* = 0.757) [19]. Additionally, patients on chronic β-blocker therapy had a lower risk of major cardiac events (odds ratio, 0.81; 95% *CI*, 0.72 to 0.91; *P* = 0.007) [19]*.*

β-blockers in the acute setting are only protective if there is “tachycardia.” They do not encounter a “self-effect” independent of the patient’s situation [20]. To attribute confidence of recommendation to the use of these drugs in critically ill patients, further randomized controlled trials (RCTs) are needed to answer questions regarding patient selection, the choice of drug, timings, and optimal hemodynamics targets.

#### In patients with septic shock, the use of β-blockers for treating persistent tachycardia may be considered

The distinction between secondary (induced by a low SV) and non-secondary (generated by an excessive sympathetic response or arrhythmic disorder) tachycardia is therefore the main element to identify patients who may benefit from β-blocker therapy.

In the first phase of septic shock, uncontrolled inflammation causes vasodilation and an increase in capillary permeability with a reduction in cardiac output (CO), which correlates with absolute and relative hypovolemia. The sympathetic hyperactivation leads to the increase in heart rate (HR), which is the primary compensatory mechanism for maintaining CO despite the reduction in preload; however, its efficacy may be blunted in those patients who are on chronic treatment with β-blockers. Persistent tachycardia, linked to a high dose of endogenous and exogenous catecholamines independent of a previous chronic ß-blocker therapy, is associated with a worse outcome. Identifying the subgroup of patients with a similar hemodynamic response represents, within the septic population, a target that correlates with a worse prognosis.

β-blockers reduce HR and control tachycardia, creating a match between contractile force and heart rate such as to reduce myocardial oxygen consumption [21].

In a randomized single-center clinical trial, the use of continuously infused β-blockers in patients who remain tachycardic after fluid resuscitation improved cardiovascular performance (HR, SV, systemic vascular resistances (SVR), and lactate) resulting in a reduction in the load of vasopressors without showing significant adverse reactions [22].

Furthermore, in septic patients with associated atrial fibrillation, the use of β-blockers seems to offer a more rapid control (1 h vs 6 h) compared to other drugs (i.e., amiodarone, magnesium sulfate, digoxin) [23].

Despite the absence of β1 receptors on vessels, infusion of selective β-blockers relieves sepsis-induced hyporesponsiveness to vasopressor treatment. β-blockers are thought to improve vascular responsiveness associated with a downregulation of α1 receptors. In this context, one study found an improvement of arterial elastance in patients with septic shock treated with the short-acting β-blocker esmolol, which may explain why the use of selective β-blockers is associated with reduced vasopressor demand despite the reduction in CO [23].

In a recent meta-analysis, treatment with continuously infused ultra-short-acting β-blockers—using a low initial dose and progressive escalation—has been demonstrated to improve survival (lower 28-day mortality, risk ratio, 0.68; 95% *CI*, 0.54–0.85; *P* < 0.001) in patients with sepsis and septic shock and heart rate > 95 bpm persisting 24 h after volume resuscitation [24]. Interestingly, the authors also reported lower white cell count in the β-blockers group, a finding that may be hypothesis generating on the immunomodulatory effects of β-blockers in septic patients.

In a meta-analysis analyzing 1839 papers, 14 studies (five RCTs, nine non-randomized studies) were deemed appropriate for inclusion. All included studies showed beneficial effects of β-blockers consisting in improved HR control at 1 h, though at 6 h there was no significant difference in HR control compared to other medications. Interestingly, amiodarone showed the longest time to achieve HR control [25].

In a systematic review, including six studies assessing mortality, four showed substantial benefits with the use of β-blockers [26]. However, due to the paucity of large-scale randomized controlled trials addressing this topic, more research is needed to ensure the validity and generalization of these findings.

In a randomized controlled trial, a group of patients with septic shock (77 patients) received a continuous infusion of esmolol to maintain a heart rate between 80/min and 94/min, compared to a control group (77 patients) with standard treatment over 96 h [22]. Target HRs were achieved in all patients in the esmolol group compared to those in the control group. The median area under the curve (AUC) for HR during the first 96 h was − 28/min (*IQR*, − 37 to − 21) for the esmolol group compared with -6/min (95% *CI*, − 14 to 0) for the control group with a mean reduction of 18/min (*P* < 0.001). The 28-day mortality, a secondary end point, was 49.4% in the esmolol group versus 80.5% in the control group (adjusted hazard ratio, 0.39; 95% *CI*, 0.26 to 0.59; *P* < 0.001).

#### Control of the heart rate and perioperative oxygen consumption in high-risk surgical patients should be achieved with the use of β-blocker drugs

The role of β-blockers in this setting is characterized by 20 years of controversy about the cost/benefit of their introduction in therapy close to the perioperative phase, as discussed again in the recent European Society of Cardiology’s (ESC) guidelines [27].

Although from a purely electrophysiological point of view for the control of perioperative tachycardia there exist several therapeutic options, the choice of calcium channel blockers is not supported by recent literature, and amiodarone represents the drug most used in the field of tachycardia related to perioperative atrial fibrillation.

In major noncardiac surgery, the maintenance of an (oral) chronic β-blocker therapy in the perioperative period is considered a marker of good quality of care. Their intake, started within 60 days of surgery, has proved to be effective in limiting perioperative complications, without however showing differences in the incidence of postoperative stroke compared to chronic beta-blocker treatment.

### Question III: What is the diagnostic-therapeutic strategy in the management of tachycardia in the ICU patient?

#### In the patient in the ICU, it is necessary to diagnose secondary tachycardia to be able to treat it with specific drugs

Tachycardia is a very frequent clinical condition but at the same time underestimated by clinicians. It often causes severe hemodynamic instability and reduced organ perfusion.

The patient populations most at risk are septic patients, multiple trauma patients, burn patients, and those undergoing cardiac surgery or major surgery. These patient populations are the ones most exposed to changes in the hemodynamic, immunological, metabolic, respiratory and neurologic function, and coagulative profile. The dysregulation of these systems is related to an increase in mortality*.*

#### The therapeutic approach to tachycardia should be based on heart rate reduction and heart rhythm control

In the strategy of therapeutic approach to the tachycardic patient, the first step is to diagnose the triggering cause and treat it (anemia, hypovolemia, dystonia, stress, hypoxemia, pain, pharmacological adrenergic hyperstimulation, etc.).

If tachycardia persists despite causative treatment, it becomes a pathological condition and should be pharmacologically managed.

In intensive care, tachycardia always represents a condition that requires medical attention, especially when associated with hemodynamic instability. The critically ill patient is often characterized by complex and sudden changes not only in the hemodynamic function but also in the immunological, metabolic, and coagulation asset. This inevitably implies the activation of the endogenous catecholamine system with sympathetic overstimulation, which is often related to organ damage with a consequent worsening of the prognosis and increased mortality.

Tachycardia-induced myocardial ischemia, the significant increase in myocardial energy reserve utilization, and the remodeling of the cellular and extracellular matrix are the possible pathophysiological mechanisms able to progressively deteriorate myocardial function and therefore cause hemodynamic instability resulting in reduced organ perfusion.

#### The administration of β-blockers in the critically ill patient should take place intravenously, preferably, with the use of rapid kinetic drugs; the transition to other routes of administration should be performed when enteral administration seems feasible

There are no studies that specifically addressed the issue of β-blockade administration strategy in patients in intensive care. However, it is the consent opinion of the working group that given the fact that the critically ill patient may present with clinical instability both in terms of hemodynamics and organ functions, the continuous intravenous administration of a short half-life beta-blocker would allow full control of the pharmacological effects and avoid adverse effects from accumulation.

Furthermore, β-blockers with high cardioselectivity should be used in critically ill patients to minimize possible hypotensive effects and the impact on organ functions (for instance, respiratory, endocrine).

Esmolol and landiolol reduce heart rate by depressing atrioventricular node conduction and reducing myocardial oxygen demand. Their pharmacokinetics allows to “titrate” the dosage and to ensure rapid adjustments on different steady-state levels of β-blockade.

The short duration of action is linked to the rapid enzymatic hydrolysis via plasma esterases; therefore, their use is indicated in patients in whom β-blockade seems to be of clinical benefit, but they must be carefully titrated to their effect: insensitivity to the context plays a central role which makes these drugs interesting in modulating the hemodynamics of the septic patient. Esmolol and landiolol have a high cardioselectivity (around 8 times higher for landiolol, 255:1 vs 33:1, respectively) associated with an ultra-short plasma half-life and defined context insensitive (greater for esmolol 9 min vs 4 min).

Metoprolol, another β-1 selective β-blocker, can be used intravenously. Slow or additional boluses can be given, and it possibly moved on to the enteral route once the clinical situation has stabilized, with no further risk of shock, and if the need arises [28].

In the septic patient, in the presence of an adequate preload, the reduction of heart rate can improve arterial elastance and allow an improvement in myocardial efficiency by ensuring systemic perfusion, which is central to the treatment. From this perspective, the difference between systolic and dicrotic notch pressure (SDP) has been proposed as an identifying parameter of those patients in whom the reduction of HR is associated with hemodynamic worsening [29]. A threshold value of SDP below 35 mmHg is considered predictive of hemodynamic worsening following the reduction of heart rate.

Therefore, the complex hemodynamic response to β-blocker therapy requires a slow and continuous adaptation to ensure the safest cardiovascular stability.

#### In the hemodynamic stable patient on chronic  β-blocker therapy, the  β-blocker treatment should not be stopped.

The recent ESC guidelines have confirmed that in surgical patients on chronic therapy with β-blockers, the treatment should not be interrupted in the perioperative period [27].

This rationale is based on numerous studies that have shown an increase in postoperative mortality in surgical patients in whom chronic β-blockers therapy was interrupted before surgery and a possible twofold increased risk of the onset of atrial fibrillation in patients in whom β-blockers therapy is interrupted for 2 days in the postoperative phase [20, 30, 31].

### Question IV.  What is the management strategy for tachyarrhythmic atrial fibrillation in critically ill patients?

#### Before starting drug therapy, all reversible causes of AF (electrolyte disturbances, acid–base abnormalities, use of beta-agonist drugs, myocardial ischemia, hypovolemia, anemia, infection, pain, and agitation) must be recognized and treated if necessary

Pharmacological treatment of AF should be undertaken only after excluding reversible causes and triggers. There are no studies to support this rationale; however, the working group supports this statement based on the recent ESC guidelines [27, 31].

#### AF is defined as a chaotic cardiac rhythm disturbance with a supraventricular start, which is identified as paroxysmal when it terminates spontaneously or with intervention within 7 days of onset

The guidelines of the ESC have divided the forms of AF into 5 subgroups: first diagnosed, paroxysmal, persistent, long-standing persistent, and permanent [27, 31].

For greater completeness, in this document, reference will not be made exclusively to the paroxysmal forms of AF but also to the other forms of AF diagnosed in the critically ill patient, identifying more characterizations when possible [18, 21] (E-Table 2—Additional File 1).

#### Regardless of the first “hemodynamic” approach, complex critically ill patients may benefit from the involvement of a multidisciplinary team that includes the referring specialists, as well as the general practitioner (for post-discharge assistance) and the family members/caregivers

In consideration of the extreme complexity implied by AF, the guidelines of the ESC suggest a multidisciplinary approach aimed also at the follow-up of the patient after discharge from a critical area [27, 31].

Regardless of acute management, from a primarily hemodynamic point of view, the management of the patient with AF provides for an in-depth clinical evaluation that includes risk and predisposing factors, the risks of stroke, the indications for anticoagulant therapy independently (or not exclusively derived) outcome of acute management, and/or interventional cardiology procedures (e.g., atrial appendage occlusion, atrial node ablation, ventricle with pacemaker implant).

#### Pharmacological treatment of AF in the critically ill patient is reserved for the hemodynamically stable patient, in whom heart rate control should be considered as the primary target

Rhythm control strategy refers to attempts to restore and maintain sinus rhythm. It may involve a combination of therapeutic approaches including synchronized electrical cardioversion, the administration of antiarrhythmic drugs, and the application of interventional cardiology procedures (e.g., transcatheter ablation) in combination with adequate control of the mean ventricular rate (rate control vs rhythm control) and the management of anticoagulant therapy [27, 31].

#### Electrical cardioversion is recommended in hemodynamically unstable patients

In the patient with hemodynamic instability (e.g., hypotension/hypoperfusion/myocardial DO_2_-VO_2_ mismatch), a DC electrical synchronized cardioversion should be performed as it is more effective than pharmacological cardioversion and results in immediate restoration of sinus rhythm [31]. Pretreatment with antiarrhythmic drugs may improve the efficacy of elective electrical cardioversion [31].

#### Heart rate control in critically ill patients with AF and normal or moderately depressed left ventricular function should be achieved with β-blockers and calcium channel blockers. In the case of EF < 40%, β-blockers and digoxin should be used

There are no clinical studies that have investigated which is the drug of first choice to treat AF in the critically ill patient. However, recent ESC guidelines recommend (IB) to use β-blockers and calcium channel blockers as first-line drugs for acute ventricular rate control in patients with AF and ejection fraction > 40% [27, 31].

In the case of EF < 40%, β-blockers and digoxin (IB) should be used, always taking into account the negative inotropic effect of the former, according to ESC guidelines 2022.

β-blockers are often first-line agents for rate control especially in the acute phase such as in the paroxysmal forms of the patient admitted to the intensive care unit [22]*.*

The choice of the drug depends on symptoms, comorbidities, and potential side effects [32, 33].

Two studies report a lower mortality associated with the use of β-blockers compared to amiodarone; one study, which included 39,693 septic patients, 60% of whom were admitted to the intensive care unit, following adjustment for confounding factors showed a relative risk of 0.67 (95% *CI*: 0.59–0.77) [34, 35].

A second study, involving patients admitted to the intensive care unit, showed a statistically nonsignificant higher mortality in patients treated with amiodarone (40%) compared with patients treated with metoprolol [36, 37].

In the acute setting, the clinician must always consider the cause or co-causes (trigger effects) of the AF (for example, anemia, infections). β-blockers and calcium channel blockers are preferred over digoxin due to their faster effect and greater efficacy even in conditions of hypertonicity of the sympathetic nervous system [27, 31]. Selectivity, especially in the patient with reduce LV systolic function, can play a role in the choice of the drug. However, studies specifically addressing this aspect in the critically ill are still needed.

Studies that have compared the different categories of drugs are burdened by a substantial risk of bias in relation to the study design, the analysis of the results, or the heterogeneity of the patients and of the dosages and methods of administration [31].

#### When drug therapy is indicated, the drug of first choice in the critically ill patient should be a rapidly acting and clearing β-blocker

The possible presence in the critically ill patient of conditions such as hepatic and renal dysfunction, preexisting cardiac dysfunction (e.g., chronic systolic dysfunction, diastolic dysfunction associated with chronic systemic arterial hypertension), or new onset (septic cardiomyopathy, perioperative ischemic myocardial injury) would make a rapid-acting and elimination β-blockers the drug of first choice [31, 38].

Furthermore, the use of a selective, rapid-acting and short-lasting β-blocker in the critically ill patient offers the advantage of being able to be easily “titrated” and of obtaining rapid reversal of any side effects with suspension. In the patient with septic shock, a favorable effect has been highlighted both in terms of reduction of tachycardia and hemodynamic stability [22, 39–41].

## Conclusions

In conclusion, literature data suggest that adrenergic stress and increased heart rate are associated with organ dysfunction and increased mortality in the critically ill. β-blockers protective effects in critically ill patients have been repeatedly reported in the literature.

Their use in the acute treatment of increased heart rate requires understanding of the pathophysiology and careful differential diagnosis, as all causes of tachycardia should be ruled out and addressed first. This document is aimed to support the healthcare personnel involved in the use of β-blockers in critically ill patients by providing good clinical practice statements.

### Supplementary Information

**Additional file 1:** **E-Table 1.** Search Strategy. **E-Table 2.** Atrial Fibrillation Table. **Fig. 1.** PRISMA flow 2020.

**Additional file 2.** List of included studies. 

## Abbreviations

AF: Atrial fibrillation
AUC: Area under the curve
CI: Confidence interval
CO: Cardiac output
CQ: Clinical question
DO_2_: Oxygen delivery
ESC: European Society of Cardiology
HR: Heart rate
ICU: Intensive care unit
IQR: Interquartile range
MVO_2_: Myocardial oxygen consumption
RCTs: Randomized controlled trials
SDP: Systolic and dicrotic notch pressure
SV: Stroke volume
SVR: Systemic vascular resistances
VO_2_: Myocardial and tissue oxygen consumption
