# Supplementary material for: The role of beta-blocker drugs in critically ill patients: a SIAARTI expert consensus statement
Source: J Anesth Analg Crit Care. 2023 Oct 23;3:41. doi: 10.1186/s44158-023-00126-2 (PMC10591347; doi:10.1186/s44158-023-00126-2)
Supplement: Supplementary file 1 — Additional file 1: E-Table 1. Search Strategy. E-Table 2. Atrial Fibrillation Table. Fig. 1. PRISMA flow 2020. [file 44158_2023_126_MOESM1_ESM.docx]

**Additional File 1.**

Search strategy and PRISMAflow 2020

**E-Table 1. Search strategy.**

| **Database:** | PuBMed. |
| --- | --- |
| **Search strings:** | ("critical care"[MeSH Major Topic] OR "critical illness"[MeSH Major Topic] OR "critical care"[Title/Abstract] OR "icu"[Title/Abstract] OR "intensive care"[Title/Abstract]) AND ("tachycardia"[MeSH Major Topic] OR "atrial fibrillation"[MeSH Major Topic] OR "atrial flutter"[MeSH Major Topic] OR "tachycardia"[Title/Abstract] OR "atrial arrhythmia"[Title/Abstract] OR "atrial fibrillation"[Title/Abstract] OR "atrial flutter"[Title/Abstract]) AND ("humans"[MeSH Terms])  ("critical care"[MeSH Major Topic] OR "critical illness"[MeSH Major Topic] OR "critical care"[Title/Abstract] OR "icu"[Title/Abstract] OR "intensive care"[Title/Abstract]) AND ("adrenergic beta 1 receptor antagonists"[MeSH Major Topic] OR "adrenergic beta antagonists"[MeSH Terms] OR "beta block"[Title/Abstract] OR "beta blockade"[Title/Abstract] OR "beta blocker"[Title/Abstract] OR "acebutolol"[Title/Abstract] OR "atenolol"[Title/Abstract] OR "bisoprolol"[Title/Abstract] OR "metoprolol"[Title/Abstract] OR "nadolol"[Title/Abstract] OR "nebivolol"[Title/Abstract] OR "propranolol"[Title/Abstract] OR "carvedilol"[Title/Abstract] OR "esmolol"[Title/Abstract] OR "labetalol"[Title/Abstract] OR "sotalol"[Title/Abstract] OR "celipropol"[Title/Abstract] OR "landiolol"[Title/Abstract]) AND ("humans"[MeSH Terms])  ("adrenergic beta 1 receptor antagonists"[MeSH Major Topic] OR "adrenergic beta antagonists"[MeSH Terms] OR "beta block"[Title/Abstract] OR "beta blockade"[Title/Abstract] OR "beta blocker"[Title/Abstract] OR "acebutolol"[Title/Abstract] OR "atenolol"[Title/Abstract] OR "bisoprolol"[Title/Abstract] OR "metoprolol"[Title/Abstract] OR "nadolol"[Title/Abstract] OR "nebivolol"[Title/Abstract] OR "propranolol"[Title/Abstract] OR "carvedilol"[Title/Abstract] OR "esmolol"[Title/Abstract] OR "labetalol"[Title/Abstract] OR "sotalol"[Title/Abstract] OR "celipropol"[Title/Abstract] OR "landiolol"[Title/Abstract]) AND ("sepsis"[MeSH Major Topic] OR "shock, septic"[MeSH Major Topic] OR "septic"[Title/Abstract] OR "sepsis"[Title/Abstract]) AND ("humans"[MeSH Terms])  ("adrenergic beta 1 receptor antagonists"[MeSH Major Topic] OR "adrenergic beta antagonists"[MeSH Terms] OR "beta block"[Title/Abstract] OR "beta blockade"[Title/Abstract] OR "beta blocker"[Title/Abstract] OR "acebutolol"[Title/Abstract] OR "atenolol"[Title/Abstract] OR "bisoprolol"[Title/Abstract] OR "metoprolol"[Title/Abstract] OR "nadolol"[Title/Abstract] OR "nebivolol"[Title/Abstract] OR "propranolol"[Title/Abstract] OR "carvedilol"[Title/Abstract] OR "esmolol"[Title/Abstract] OR "labetalol"[Title/Abstract] OR "sotalol"[Title/Abstract] OR "celipropol"[Title/Abstract] OR "landiolol"[Title/Abstract]) |
| **Time restriction:** | None. |
| **Exclusion:** | Not in English, conference proceedings, case series, case reports. |
| **Inclusion:** | Original papers (any designs), narrative reviews, systematic reviews, meta-analysis, position papers, guidelines, experimental studies, randomized controlled trials. |

**Figure 1: “PRISMA flow”.**

PRISMA 2020 flow diagram

for new systematic reviews which included searches of databases and registers only.

**

**E-Table 2: “Atrial Fibrillation Table”**

| **Atrial Fibrillation** | **Definition** |
| --- | --- |
| *First diagnosis* | Previously undiagnosed AF, regardless of its duration or the presence/severity of AF-related symptoms. |
| *Paroxysmal* | AF ending spontaneously or with intervention within 7 days of onset. |
| *Persistent* | Atrial fibrillation sustained continuously for more than 7 days, including episodes aborted by cardioversion (pharmacological or electrical) after at least 7 days. |
| *Long-standing persistent* | Continuous AF lasting >12 months when a rhythm control strategy was chosen. |
| *Permanent* | AF accepted by patient and clinician, with no further attempts to restore/maintain sinus rhythm.  Permanent AF represents a therapeutic attitude of the patient and physician rather than an intrinsic pathophysiological attribute of AF, and the term should not be used in the context of a rhythm control strategy with antiarrhythmic drugs, therapy, or AF ablation. If a rhythm control strategy were adopted, the arrhythmia would be reclassified as "long-lasting AF". |
